# Current knowledge and recent advances in understanding metabolism of the model cyanobacterium *Synechocystis* sp. PCC 6803

**DOI:** 10.1042/BSR20193325

**Published:** 2020-04-03

**Authors:** Lauren A. Mills, Alistair J. McCormick, David J. Lea-Smith

**Affiliations:** 1 School of Biological Sciences, University of East Anglia, Norwich Research Park, Norwich NR4 7TJ, United Kingdom; 2 Institute of Molecular Plant Sciences, School of Biological Sciences, University of Edinburgh, Edinburgh EH9 3BF, United Kingdom; 3 Centre for Synthetic and Systems Biology, University of Edinburgh, Edinburgh EH9 3BF, United Kingdom

**Keywords:** comparative genomics, cyanobacteria, degradation, metabolism, Synechocystis

## Abstract

Cyanobacteria are key organisms in the global ecosystem, useful models for studying metabolic and physiological processes conserved in photosynthetic organisms, and potential renewable platforms for production of chemicals. Characterizing cyanobacterial metabolism and physiology is key to understanding their role in the environment and unlocking their potential for biotechnology applications. Many aspects of cyanobacterial biology differ from heterotrophic bacteria. For example, most cyanobacteria incorporate a series of internal thylakoid membranes where both oxygenic photosynthesis and respiration occur, while CO_2_ fixation takes place in specialized compartments termed carboxysomes. In this review, we provide a comprehensive summary of our knowledge on cyanobacterial physiology and the pathways in *Synechocystis* sp. PCC 6803 (*Synechocystis*) involved in biosynthesis of sugar-based metabolites, amino acids, nucleotides, lipids, cofactors, vitamins, isoprenoids, pigments and cell wall components, in addition to the proteins involved in metabolite transport. While some pathways are conserved between model cyanobacteria, such as *Synechocystis*, and model heterotrophic bacteria like *Escherichia coli*, many enzymes and/or pathways involved in the biosynthesis of key metabolites in cyanobacteria have not been completely characterized. These include pathways required for biosynthesis of chorismate and membrane lipids, nucleotides, several amino acids, vitamins and cofactors, and isoprenoids such as plastoquinone, carotenoids, and tocopherols. Moreover, our understanding of photorespiration, lipopolysaccharide assembly and transport, and degradation of lipids, sucrose, most vitamins and amino acids, and haem, is incomplete. We discuss tools that may aid our understanding of cyanobacterial metabolism, notably CyanoSource, a barcoded library of targeted *Synechocystis* mutants, which will significantly accelerate characterization of individual proteins.

## 1. Introduction

Cyanobacteria are the only prokaryotes capable of oxygenic photosynthesis. Since their appearance >2.4 billion years ago [1], cyanobacteria have profoundly impacted Earth’s climate and ecosystem, most notably in generation of an oxygenic atmosphere [2]. In the current ecosystem, cyanobacteria are a diverse phylum of photosynthetic prokaryotes that account for approximately a quarter of global carbon fixation [3] and a high proportion of marine nitrogen fixation [4,5]. Some species also show great potential as biotechnology platforms for synthesis of pharmaceuticals, industrial compounds and biofuels, due to their highly efficient conversion of water and CO_2_ to biomass using solar energy [6–8]. Others are used in the food, dye, cosmetics and nutraceutical industries with their global market projected to be worth >£1.5 billion by 2026 [9]. Certain species are also sources of natural products, including antifungal, antibacterial and anti-cancer compounds, and toxins deleterious to human and animal health [10,11]. Chloroplasts probably descend from an internalized cyanobacterium [12], thus certain physiological and biochemical features are conserved in higher photosynthetic organisms, making cyanobacteria excellent chassis for production of plant-derived natural products, like terpenes. Many key processes conserved throughout the photosynthetic lineages were first characterized in cyanobacteria [13,14], and there is significant interest in engineering cyanobacterial enzymes and CO_2_-concentrating mechanisms into crop plants [15–19].

Despite their importance, our understanding of many key features of cyanobacterial physiology and biochemistry is poor. For example, in *Synechocystis* sp. PCC 6803 (*Synechocystis*), the most widely studied cyanobacterium, less than 1200 coding sequences (∼30%) have assigned function (469 in metabolism and 115 in transport: Highlighted in red in Supplementary Table S1; ∼558 in other cellular processes (including transposons and transposon related functions): Highlighted in red in Supplementary Table S3), which is less than half compared with *Escherichia coli* [20]. Of these coding sequences, only a small proportion have been characterized in a cyanobacterium [21], with the majority of assigned functions based on studies of homologues in other bacteria, even though the function, catalytic activity and importance of characterized genes may differ significantly between phototrophic and heterotrophic bacteria. It is also likely that a proportion of these coding sequences have incorrectly assigned functions. Several examples of *Synechocystis* genes that were experimentally validated as having functions different to the original assigned function, based on homology with genes from heterotrophic bacteria, are discussed throughout the review.

In this review we will provide a detailed overview of the metabolic biochemistry and transport processes found in cyanobacteria, with a focus on the model unicellular species *Synechocystis* and to a lesser degree, *Synechococcus elongatus* PCC 7942 (*Synechococcus*). In each section we will highlight recent findings pertaining to each particular metabolic pathway, including central carbon and sugar metabolism, amino acid, nucleotide, cofactor and vitamin, lipid and membrane components, isoprenoid and pigment biosynthesis, and the transporters localized in the different membrane compartments. While many cyanobacteria are filamentous, with some incorporating heterocysts (specialized nitrogen fixing cells), describing the additional level of physiological complexity in these species is beyond the scope of this review (for an excellent recent review see [22]). Other aspects of cyanobacteria, such as photosynthesis and electron transport, have also been the subject of a recent review [23], and will not be discussed, except when electron transport chain components are involved in metabolism.

In the interests of brevity, the majority of enzymatic steps will not be mentioned in the text but outlined in subsequent figures. Steps to which an enzyme from *Synechocystis* has not been assigned are indicated by only an arrow with no abbreviated protein name in close proximity. The discussion will primarily focus on reactions that differ in cyanobacteria compared with model heterotrophs, or have been specifically investigated in model cyanobacteria. In most cases, only the abbreviated protein name is included in the text, although full names are outlined in Supplementary Table S1 (Column C). We have also incorporated four tables, to help guide future work on identifying homologues and assigning putative protein function. Supplementary Table S1 lists the *Synechocystis* proteins in each metabolic process, in the order outlined in the text. Also shown are the *E. coli* K12 proteins demonstrating the highest sequence similarity to individual *Synechocystis* proteins. Supplementary Table S2 is in the opposite format, and includes a list of *E. coli* K12 proteins with assigned functions, and the *Synechocystis* proteins with the highest homology to each *E. coli* protein. Supplementary Table S3 includes a list of *Synechocystis* proteins potentially involved in processes other than metabolism and transport, while Supplementary Table S4 includes all remaining *Synechocystis* proteins that have no assigned function. We will also highlight the aspects of cyanobacterial physiology and biochemistry that have yet to be elucidated and some tools in development, most notably CyanoSource, a mutant library and plasmid resource for *Synechocystis*, which will accelerate research efforts in this field.

## 2. The physiology of *Synechocystis* sp. PCC 6803

**Figure 1:**
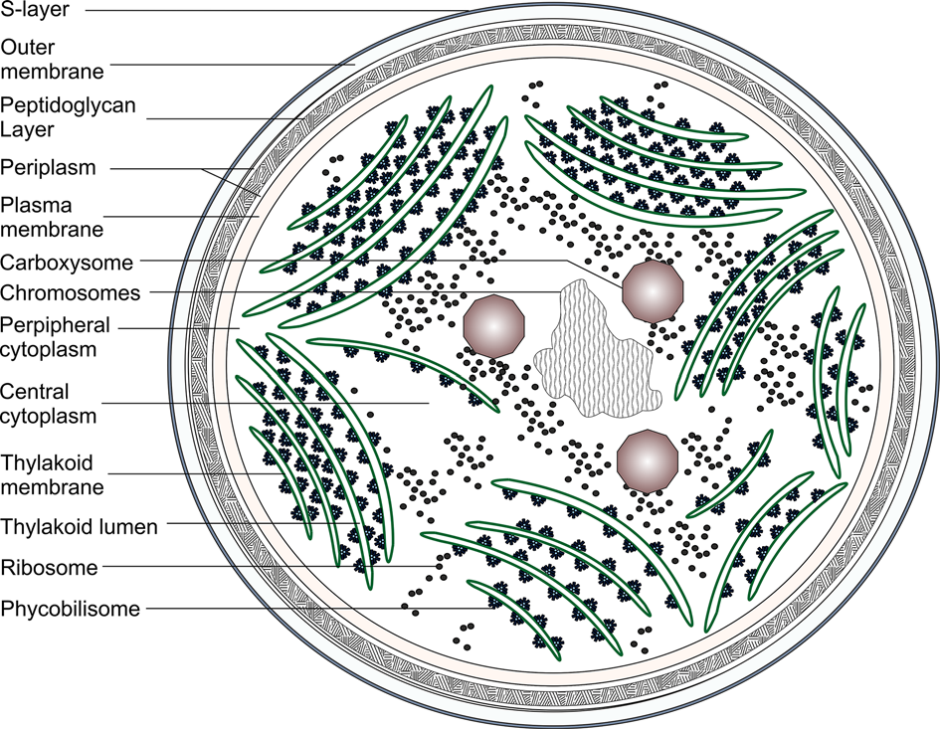
Schematic detailing the ultrastructure of *Synechocystis* sp. PCC 6803 showing various subcellular components Schematic adapted from [32,34].

In order to understand cyanobacterial metabolism, it is first necessary to describe their physiology, which is more complex than most other prokaryotes. The majority of cyanobacterial species incorporate an array of internal thylakoid membranes (TM) enclosing the thylakoid lumen, in addition to a cell envelope consisting of the plasma membrane (PM), peptidoglycan layer and outer membrane (OM) [24] (Figure 1). In *Synechocystis* and some other cyanobacteria, the S-layer, a paracrystalline protein layer, surrounds the OM [25]. TMs may contain perforations allowing transport of molecules or proteins through the array [26]. Cytoplasmic localized compartments such as the carboxysome, the site of carbon fixation, and various storage bodies accumulating glycogen, cyanophycin, polyhydroxybutyrate, lipids and polyphosphate, are predominantly distributed in the central area of the cell [27,28].

Only the primordial cyanobacterial species, *Gloeobacter kilaueensis* JS1 and *Gloeobacter violaceus* PCC 7421, both of which are extremely slow growing, lack TMs [29,30]. Therefore, there must be clear advantages in incorporating a series of internal membranes. The most obvious is the increased area available to accommodate photosynthetic complexes, in addition to incorporating a compartment that can be optimized for specialized functions. In *Synechocystis*, it has been demonstrated that the majority of characterized TM localized proteins are involved in photosynthetic and respiratory energy generation, suggesting that this is the primary function of this compartment [31,32]. In turn, these advantages must outweigh potential burdens arising from the additional complexity imposed on the cell. These burdens include the requirement for specialized cellular systems to target proteins and metabolites to the correct compartment, organize and pack TMs within the cell, and to partition TMs between daughter cells during division.

In *Synechococcus*, TMs are arranged in orderly sheets parallel to the PM with areas of convergence between the two compartments at various points [33]. Whether the TM and PM are two separate compartments is yet to be confirmed. TM arrangement in *Synechocystis* is more complicated with individual sheets often displaying disparate patterns. Three-dimensional imaging demonstrates that the majority of TMs arrange in stacks of parallel sheets that converge in distinct structures near the PM [34]. However, in contrast with earlier reports, the thylakoid and plasma membranes were shown to be two separate compartments, although the distance between them was sometimes as little as 2 nm. This suggests that processes occurring in the two compartments are spatially separated. A dense material was observed between this junction that may play a role in ‘attachment’ of the thylakoids to the cell wall but the exact process and the proteins/compounds involved, has not been determined.

## 3. Central metabolism

**Figure 2:**
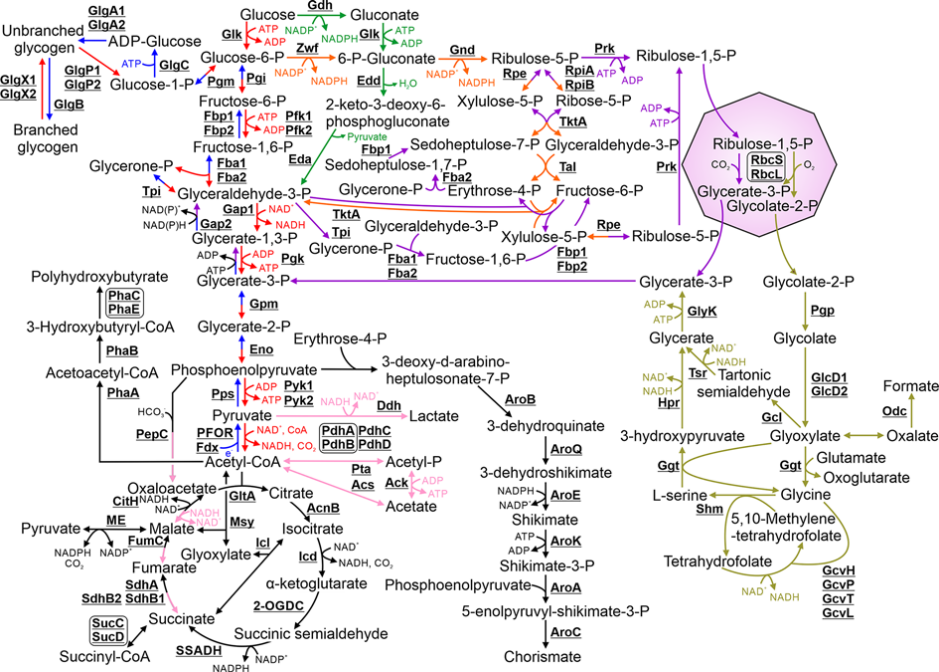
Schematic detailing the pathways involved in central metabolism Biosynthetic steps involved in glycolysis and gluconeogenesis are highlighted in red and blue respectively. Steps in the Entner–Doudoroff pathway are highlighted in green. Steps involved in the oxidative pentose phosphate pathway and the Calvin–Benson–Bassham cycle are highlighted in orange and purple, respectively. Fermentation pathways are highlighted in pink. Photorespiration pathways are highlighted in olive. Where enzymes catalyse reactions in two pathways, the arrows are split between their respective colours. The carboyxsome is represented as a purple octagon. Cofactors in each reaction are shown with the exception of protons, water, oxygen and inorganic phosphate.

In this review, cyanobacterial central metabolism will include glycolysis/gluconeogenesis, the tricarboxylic acid (TCA) cycle, the pentose phosphate (PP) pathway and the Calvin–Benson–Bassham (CBB) cycle, including carbon fixation, in addition to pathways for production of storage compounds, fermentation products and chorismate, a key intermediate for other pathways (Figure 2). Many enzymes involved in these pathways are conserved between *Synechocystis* and *E. coli* (Supplementary Table S1). Therefore, research related to protein function has primarily focused on the processes and enzymatic steps that differ in cyanobacteria compared with model heterotrophs.

### 3.1 Catabolism of glucose and glycogen

Carbon based inputs into central metabolism can be derived from carbon fixation, catabolism of glycogen or via import of glucose. The ability to import glucose enables some cyanobacteria, including certain *Synechocystis* substrains, to grow heterotrophically or mixotrophically [35]. Glucose is imported into the cell via the transporter, GlcP [36]. There are three proposed degradation pathways, which may be active under different environmental conditions [37]. Enzymes in the first two, glycolysis (the Embden–Meyerhof–Parnas (EMP) pathway) and the oxidative PP pathway, are generally highly conserved between *Synechocystis* and *E. coli* (Supplementary Table S1), and consequently these processes have not been extensively investigated in cyanobacteria. However, there are some differences and additional enzymes found in cyanobacteria. For example, homology between the *Synechocystis* and *E. coli* PdhA and PdhB subunits of pyruvate dehydrogenase is low (E value = 0.007 and 5.66E-04, respectively), and this complex has not been characterized in a cyanobacterium. *Escherichia coli* encodes only a class II fructose-1,6-bisphophosphate aldolase (Fbp2) for glycolysis, while *Synechocystis* also encodes a class I isoform (Fbp1). While the role of Fbp1 has not been determined in *Synechocystis*, expression of Fbp1 from the cyanobacterium *Halothece* sp. PCC 7418 in *Synechococcus* has been demonstrated to confer salt tolerance on this species [38]. The *Synechocystis* genome also encodes a protein, OpcA, which is not present in *E. coli*, and has been suggested to be key for glucose-6-phosphate dehydrogenase (Zwf) activity, the first step of the oxidative PP pathway [39]. However, glucose-6-phosphate dehydrogenase activity was similar to wild-type when OpcA was deleted in *Synechocystis* [40]. Recently, a third glycolytic pathway was identified in *Synechocystis* (the Entner–Doudoroff (ED) pathway) [37]. This pathway allows conversion of glucose to the oxidative PP intermediate 6-P-gluconate, which is then converted into glyceraldehyde-3-P. The ED pathway is required for optimal photoautotrophic growth and glycogen catabolism, and possibly also optimal activity of the CBB cycle [41].

### 3.2 Carbon fixation and the Calvin–Benson–Bassham cycle

As the enzymes of the CBB cycle are not isolated in a sub-cellular organelle as in eukaryotes (i.e. the chloroplast), some reactions are shared with EMP and OPP pathways. The CBB cycle can be divided into two stages: (1) conversion of ribulose-1,5-P and CO_2_ into two molecules of glycerate-3-P via ribulose-1,5-P carboxylase/oxygenase (RuBisCO), which is located in carboxysomes; (2) regeneration of the precursor, ribulose-1,5-P, consuming ATP and NADPH predominantly derived from photosynthesis. The requirement to regenerate ribulose-1,5-P leads to one major difference in the EMP pathway between cyanobacteria and heterotrophs. In *E. coli*, glyceraldehyde-3-P dehydrogenase (Gap) catalyses the reversible oxidative phosphorylation of glyceraldehyde-3-P to glycerate-1,3-P, resulting in interconversion between NAD^+^ to NADH. In contrast, *Synechocystis* Gap1 displays only glycolytic activity and a strict affinity for NAD^+^. A second isoform, Gap2, catalyses the reverse reaction required for the CBB cycle using NADH and potentially also NADPH, which is generated in large amounts via photosynthesis [42].

### 3.3 Photorespiration

RuBisCO can assimilate O_2_ instead of CO_2_, resulting in the production of one molecule each of glycerate-3-P and glycolate-2-P. The latter product is toxic to chloroplast metabolism in photosynthetic eukaryotes and likely also to *Synechocystis* at high concentrations [43]. Therefore, glycolate-2-P is converted into glycerate-3-P via the photorespiratory salvage pathway, a multi-step process conserved in most organisms that perform oxygenic photosynthesis [44]. Glycolate-2-P is first converted into glyoxylate by GlcD1 or GlcD2. Three subsequent photorespiratory pathways for catabolism of glyoxylate have been proposed in *Synechocystis* and deletion of genes in each pathway results in a mutant that requires high CO_2_ conditions for survival [43]. The first involves conversion of glyoxylate to glycerate-3-P via tartonic semialdehyde biosynthesis, the second, conversion of glyoxylate to glycerate-3-P via glycine and L-serine interconversion, and the third conversion of glyoxylate to oxalate, which is subsequently converted into formate. The enzymes involved in several of these pathways have been predominantly identified in *Arabidopsis thaliana*, with putative homologs present in cyanobacteria [45]. Of these, Shm, involved in the second pathway, and GlcD1, have been shown to display similar enzymatic activity to their *A. thaliana* homologs [45]. Deletion of GlcD1 and GlcD2 in *Synechocystis* results in a complete loss of photorespiratory activity [43]. However, the role of the other putative cyanobacterial homologs has not been determined and many proteins currently assigned to photorespiration, as outlined in Eisenhut et al. [43], have been suggested to catalyse alternative reactions. Moreover, in the third pathway, only one putative enzyme, Odc, has been identified.

### 3.4 Synthesis of carbon storage compounds

Cyanobacteria require carbon storage compounds for periods when photosynthesis is not sufficient for the cells energy and metabolic requirements. In *Synechocystis*, under conditions where cells are accumulating excess sugars, a high proportion of glycerate-3-P generated via CO_2_ fixation is converted into glycogen (reviewed in [46]). In *E. coli*, ADP-glucose is used as the substrate to generate the primary, unbranched polymer via GlgA. However, two GlgA isoforms are present in *Synechocystis* with likely roles in elongating the polymer at varying length [47]. Glycogen catabolism in *Synechocystis* is catalysed by two isoforms of GlgX (GlgX1 and GlgX2) and GlgP (GlgP1 and GlgP2). The role of GlgX1 and GlgX2 has not been determined. The GlgP proteins perform the same catalytic activity under different environmental conditions, cleavage of glycogen to individual glucose-1-P residues [48]. When *Synechocystis* is exposed to certain stress conditions, an additional carbon storage compound, the polymer polyhydroxybutyrate, is synthesized from acetyl-CoA via PhaA, PhaB, and the PhaC/PhaE complex [49–51].

### 3.5 The tricarboxylic acid cycle

The tricarboxylic acid (TCA) cycle differs in cyanobacteria compared with heterotrophic bacteria, as highlighted by recent work in the last decade. Cyanobacteria lack the enzyme α-ketoglutarate dehydrogenase, which catalyses the fourth step of the TCA pathway in *E. coli*: conversion of α-ketoglutarate to succinyl-CoA. Instead, some cyanobacteria, including *Synechocystis*, have genes encoding two enzymes, α-ketoglutarate decarboxylase (2-OGDC) and succinic semialdehyde dehydrogenase (SSADH), which convert α-ketoglutarate into succinic semialdehyde, then succinic semialdehyde into succinate, respectively [52]. Compared with the standard TCA cycle, where conversion of α-ketoglutarate to succinate results in production of one NADH and one GTP, the 2-OGDC/SSADH pathway results in production of one NADPH [52]. Only the soluble subunits of succinate dehydrogenase, catalysing the sixth step, have been identified in cyanobacteria [23]. Succinate dehydrogenase is integrated into the thylakoid membrane interlinked photosynthetic and respiratory electron chain [53]. *Synechocystis* also encodes a succinyl-CoA synthetase complex (SucC/SucD), which probably catalyses the reversible conversion of succinate into succinyl-CoA in cyanobacteria [54], required for biosynthesis of methionine and lysine. Several recent papers have investigated the enzymatic properties of TCA enzymes conserved between cyanobacteria and heterotrophic bacteria [55–57]. In contrast with many heterotrophic bacteria, *Synechocystis* citrate synthase (GltA) was shown only to catalyse generation of citrate, not its cleavage. *Synechocystis* GltA has a lower substrate affinity and turnover rate than the *E. coli* homologue, is not inhibited by ATP and NADH, but is inhibited by phosphoenolpyruvate [55].

### 3.6 Alternate biosynthetic pathways linking metabolites of the tricarboxylic acid cycle, photorespiration and glycolysis

A range of additional pathways link the TCA cycle with glycolysis and photorespiration. Glyoxylate, produced via photorespiration, also plays a role in the glyoxylate cycle. This cycle consists of three TCA enzymes and two additional enzymes unique to this pathway: the first, isocitrate lyase (Icl), converts the TCA cycle intermediate isocitrate to succinate and glyoxylate; the second, malate synthase (Msy), converts glyoxylate and acetyl-CoA to the TCA cycle intermediate, malate. While activity of glyoxylate cycle enzymes has been detected in some cyanobacteria (reviewed in [58]), it is unclear whether *Synechocystis* encodes active variants of Icl and Msy.

Phosphoenolpyruvate carboxylase (PepC) catalyses the conversion of phosphoenolpyruvate, a glycolysis intermediate, and HCO_3_^−^ to oxaloacetate, a TCA intermediate [59]. PepC can therefore be considered an inorganic carbon fixing enzyme (i.e. akin to RuBisCO). Metabolic flux analysis has shown that as much as 25% of all inorganic carbon fixation occurs via PepC in *Synechocystis* cultured under mixotrophic or heterotrophic conditions [60]. An additional protein, malic enzyme (ME), catalyses the reversible conversion of malate, a TCA intermediate, and pyruvate [61]. Deletion of ME in *Synechocystis* results in a mutant that displays poor growth when exposed to continuous but not diurnal light [62]. It was hypothesized that ME is required for pyruvate biosynthesis under continuous light.

### 3.7 Fermentation pathways

Three possible fermentation pathways are present in *Synechocystis* that generate D-lactate, acetate or succinate, respectively. Presumably fermentation plays a role in energy generation when cyanobacteria are exposed to long periods of darkness under anoxic conditions, but the importance of these pathways during changing environmental conditions has not been determined. D-lactate, acetate and succinate production has been observed in wild-type *Synechocystis* cells but only after 3 days growth under dark, anaerobic conditions [63]. A homolog of lactate dehydrogenase (Ddh), which converts pyruvate and NADH to lactate and NAD^+^, is encoded by *Synechocystis*. Two possible pathways for acetate production may be present in *Synechocystis*: (1) Conversion of acetyl-CoA to acetyl-P, then acetate, via phosphotransacetylase (Pta) and acetate kinase (Ack), respectively; (2) Direct reversible conversion of acetyl-CoA to acetate via acetyl-CoA synthetase (Acs) [63]. Production of succinate relies primarily on phosphoenolpyruvate as the initial substrate, which is subsequently converted to oxaloacetate via PepC and then fed into the reverse TCA cycle [64].

### 3.8 Chorismate biosynthesis

Chorismate is the precursor for biosynthesis of a range of amino acids and cofactors, and has further importance in cyanobacteria as the substrate for production of phylloquinone, plastoquinone, phenylalanine, tyrosine, folate and molybdopterin, in addition to tocopherols and carotenoids. The glycolytic and PP pathway intermediates phosphenolpyruvate and erythrose-4-P are the substrates for production of chorismate via a 7-step pathway in *E. coli*. However, the enzyme catalysing the first step, condensation of phosphoenolpyruvate and erythrose-4-P, has not been identified in *Synechocystis* [65]. *Synechocystis* proteins demonstrating high sequence similarity to five other enzymes in the *E. coli* pathway have been identified (Supplementary Table S1) with the exception of the third enzyme, AroQ (No BLAST match). It is unclear from the literature how function was assigned to *Synechocystis* AroQ, encoded by *sll1112* in the KEGG database.

## 4. Metabolism and degradation of nucleotide sugars and sugar osmolytes

**Figure 3:**
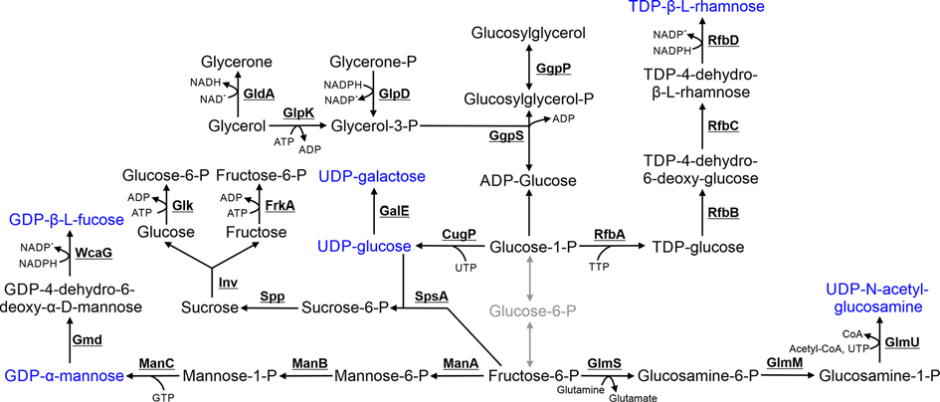
Metabolism and degradation of nucleotide sugars and sugar osmolytes Compounds highlighted in blue are substrates for lipopolysaccharide biosynthesis. Steps highlighted in grey are compounds and reactions not involved in these pathways but detailed in Figure 1. Cofactors in each reaction are shown with the exception of protons, water, oxygen and inorganic phosphate.

A range of nucleotide sugars required for lipopolysaccharide (LPS) biosynthesis or as cofactors for other reactions (i.e. UDP-glucose) are synthesized by *Synechocystis* (Figure 3). LPSs contain a range of sugar residues including rhamnose, galactose, glucosamine, mannose and fucose, which in *Synechocystis* are incorporated as 2,3-di-methyl-fucose and 2-methyl-fucose. 2-Methylxylose has also been reported in *Synechocystis* [66]. Only some of the biosynthetic pathways synthesizing the LPS sugar precursors have been identified in cyanobacteria, although predominantly on the basis of identifying proteins with high sequence similarity to characterized enzymes from heterotrophic bacteria. TDP-β-L-rhamnose is synthesized by a four step pathway from glucose-1-P. There are two potential homologs in *Synechocystis* for each of the last three enzymes in the pathway, RfbB, RfbC and RfbD, but the function of these isoenzymes has not been determined. UDP-N-acetylglucosamine is synthesized by a three step pathway from fructose-6-P and is the precursor not just for LPSs but also peptidoglycan. UDP-glucose is synthesized from glucose-1-P by CugP, a non-GalU UDP-glucose pyrophosphorylase, which differs from the GalU UDP-glucose pyrophosphorylase reaction conducted in most proteobacteria, including *E. coli* [67]. A UDP-glucose 4-epimerase (GalE) then catalyses the conversion of UDP-glucose to UDP-galactose. GDP-mannose is synthesized from fructose-6-P by a three step reaction and GDP-fucose from GDP-mannose by a two-step pathway. None of the proteins in these pathways have been characterized in cyanobacteria although deletion of the last gene in this pathway in *Synechocystis*, WcaG, resulted in production of carotenoids lacking fucose [68].

Several sugars act as osmolytes, notably sucrose and glucosylglycerol. Osmolytes play a role in *Synechocystis* in salt tolerance [69,70]. In *Synechocystis*, sucrose is synthesized from UDP-glucose (or ADP-glucose) and fructose-6-P by two enzymes: SpsA and Spp [71,72]. Sucrose breakdown in *Synechocystis* is catalysed by an invertase (Inv) [73], resulting in production of glucose and fructose, which are likely phosphorylated to glucose-6-P by Glk and fructose-6-P by FrkA, and cycled back into glycolysis. A putative glucose kinase and fructose kinase are encoded in the *Synechocystis* genome, but have not been characterized. Glucosylglycerol is synthesized from ADP-glucose and glycerol-3-P via two enzymes: GgpS and GgpP [74]. Glycerol-3-P is derived from either the TCA cycle intermediate glycerine-3-P or possibly imported.

## 5. Amino acid biosynthesis and degradation

**Figure 4:**
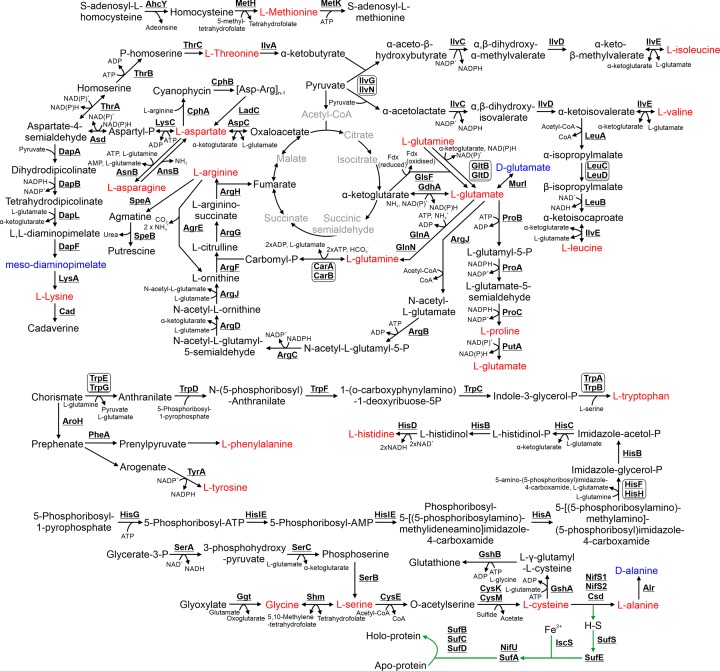
Metabolism of amino acids, cyanophycin, glutathione and iron–sulfur clusters The 20 L-amino acids are highlighted in red while amino acids incorporated into peptidoglycan are highlighted in blue. The iron–sulfur biosynthetic pathways is highlighted in green. Steps highlighted in grey are compounds and reactions not involved in these pathways but detailed in Figure 1. Cofactors in each reaction are shown with the exception of protons, water, oxygen and inorganic phosphate.

*Synechocystis* synthesizes 20 L-amino acids and two D-amino acids (Figure 4). The majority of enzymes involved in amino acid biosynthesis display high sequence similarity between *Synechocystis* and *E. coli* (Supplementary Table S1). Amino acids are synthesized from a range of substrates, including pyruvate, the TCA cycle intermediates α-ketoglutarate and oxaloacetate, chorismate, the nucleotide intermediate, 5-phosphoribosyl-1-pyrophosphate (discussed in section 6), and glycerate-3-P or glyoxylate. Biosynthesis of amino acids is divided into sections below based on the substrates utilized.

### 5.1 Isoleucine, valine and leucine biosynthesis

α-Ketobutyrate (synthesized from L-threonine by IlvA) and pyruvate are the substrates for biosynthesis of L-isoleucine, while pyruvate is the sole substrate for L-valine and L-leucine biosynthesis. The enzymatic steps in *Synechocystis* are similar to those in *E. coli*, with the exception of the first step. In *E. coli* biosynthesis of α-acetolactate and α-aceto-β-hydroxybutyrate are typically catalysed by the IlvB/IlvN complex. However, in *Synechocystis*, the homologue for IlvB was identified as 2-OGDC in the TCA cycle (Section 3.5) [52]. An alternate acetolactate synthase, IlvG, demonstrates high sequence similarity to *E. coli* IlvG (*E* value = 0). IlvG may form a complex with IlvN and catalyse this step [75] but this requires further verification.

### 5.2 Glutamate, glutamine and proline biosynthesis

The TCA cycle intermediate α-ketoglutarate is the substrate for L-glutamate biosynthesis which in turn is the substrate for production of L-glutamine, D-glutamate and L-proline. D-glutamate is synthesized by MurI and is incorporated into peptidoglycan. Two different glutamine synthetases, GlnA and GlnN, convert L-glutamate into L-glutamine [76], and in the process incorporate ammonia into amino acid biosynthesis. Alternatively, several enzymes catalyse the opposite reaction where L-glutamine is converted into L-glutamate, including an NAD(P)H or possibly ferredoxin-dependent glutamate synthase (GltB/GltD) and a ferredoxin-dependent glutamate synthase (GlsF) [77]. L-proline is synthesized via three enzymes (ProA, ProB, ProC). *Synechocystis* also encodes a putative proline oxidase, PutA, which catabolized L-proline to L-glutamate, reducing NADP^+^ and possibly a quinone in the process [78].

### 5.3 Arginine biosynthesis

L-arginine is synthesized from L-glutamate via eight enzymatic steps, the sixth requiring carbomyl-P, which is synthesized from L-glutamine via CarA/CarB. This pathway is very similar to that in *E. coli*. However, *Synechocystis* does not encode ArgA or ArgE, catalysing the first and fifth steps of the pathway. Instead, it encodes ArgJ, a bifunctional enzyme that catalyses both these enzymatic reactions. Recently, an ornithine-ammonia cycle was identified in *Synechocystis* [79]. This cycle utilizes ArgF, ArgG, ArgH, and an additional enzyme, AgrE. AgrE converts L-arginine into L-ornithine, releasing ammonia in the process [80]. *Synechocystis* also encodes two putative SpeA and two putative SpeB proteins, which play a role in degradation of L-arginine to putrescine, a polyamine. In *E. coli*, putrescine can be used as a nitrogen and carbon source via conversion to succinate [80]. Whether putrescine has a similar role in cyanobacteria has not been determined.

### 5.4 Aspartate, cyanophycin and lysine biosynthesis

L-aspartate is synthesized from oxaloacetate and L-glutamate by AspC. L-aspartate and L-arginine are the substrates for cyanophycin, a nitrogen storage polymer. Cyanophycin is synthesized by CphA and then converted back to L-aspartate and L-arginine by CphB and LadC [81]. L-aspartate is converted into aspartate-4-semialdehyde, which is the substrate for biosynthesis of L-threonine and L-lysine. *Synechocystis* encodes all the enzymes in the five step diaminopimelate aminotransferase pathway required for L-lysine biosynthesis [82,83]. The third reaction, conversion of tetrahydrodipicolinate to L,L-diaminopimelate, is catalysed by DapL. In contrast, *E. coli* requires three enzymes, DapC, DapD and DapE, for this conversion. L-lysine is the substrate for production of the siderophore cadaverine by Cad. Three enzymes, ThrA, ThrB and ThrC, convert aspartate-4-semialdehyde into L-threonine by a pathway similar to that in *E. coli*.

### 5.5 Methionine biosynthesis

In *E. coli*, L-methionine is also synthesized from aspartate-4-semialdehyde. However, the *Synechocystis* genome does not encode homologues to MetA, MetB or MetC (Supplementary Table S2), the first three enzymes in the pathway. However, the genome does encode a putative MetH enzyme, which catalyses the last step, conversion of homocysteine to L-methionine. The enzymatic steps prior to this have not been determined, nor has the original substrate from which L-methionine is synthesized. The *Synechocystis* genome also encodes a putative MetK enzyme, which converts L-methionine into S-adenosyl-L-methionine, a cofactor utilized in many other reactions, most notably in biosynthesis of cyanocobalamin (Vitamin B_12_; Section 10.4). A putative AhcY enzyme is also encoded, which converts S-adenosyl-L-homocysteine, the product of reactions which use S-adenosyl-L-methionine as a cofactor, back to homocysteine.

### 5.6 Tryptophan, phenylalanine and tyrosine biosynthesis

Chorismate is the substrate for L-tryptophan, L-phenylalanine and L-tyrosine biosynthesis. The majority of enzymes involved in L-tryptophan biosynthesis are highly conserved between *E. coli* and *Synechocystis*. Attempts to generate an auxotrophic mutant of TrpB, one of the subunits catalysing the final step of L-tryptophan biosynthesesis, were unsuccessful [84], suggesting that it cannot be imported from the external environment. The pathway for L-phenylalanine and L-tyrosine biosynthesis differs between the two species and has not been completely determined in cyanobacteria. Both amino acids are synthesized from prephenate. However, only the second step of tyrosine biosynthesis, conversion of arogenate to L-tyrosine, has been determined, although sll1662 (PheA) has been speculated to catalyse the first step of L-phenylalanine biosynthesis, conversion of prephenate to prenylpyruvate [85].

### 5.7 Histidine biosynthesis

L-histidine, synthesized from the nucleotide precursor, 5-phosphoribosyl-1-pyrophosphate, is synthesized via a nine-step pathway in *E. coli*. Proteins demonstrating high sequence similarity to all characterized histidine biosynthetic enzymes in *E. coli* have been identified in *Synechocystis*. However, there are two putative HisC and HisD enzymes in *Synechocystis*. The function of these isoenzymes has not been determined.

### 5.8 Serine, glycine, cysteine and alanine biosynthesis

L-serine can potentially be synthesized via two routes. The first is via a three step light-independent pathway, which has been characterized in *Synechocystis* [86]. However, the second enzyme in this pathway, SerC has also been suggested to catalyse the transanimation reaction in photorespiration (Section 3.3) [43]. In the second pathway, L-serine (and also glycine) is synthesized from glyoxylate via the photorespiratory pathway or glyoxylate cycle in those species that encode the relevant enzymes. L-cysteine is then produced from L-serine via a two step pathway, the second of which could potentially be catalysed by either CysK or CysM. L-cysteine is subsequently desulfonated to produce L-alanine by Csd [87], which is subsequently converted to D-alanine, a component of peptidoglycan, via Alr.

### 5.9 Glutathione biosynthesis

L-cysteine and L-glutamate are the substrates for the first step of glutathione biosynthesis. Glutathione is a thiol that plays a key role in metal detoxification and tolerance of oxidative stress in *Synechocystis* [88]. The first step of glutathione biosynthesis is catalysed by GshA, encoded by an essential gene in *Synechocystis* [89]. In contrast, the enzyme catalysing the second step, GshB is non-essential, suggesting that glutathione is not required for *Synechocystis* viability but that the precursor, L-γ-glutamyl-L-cysteine, is [89].

### 5.10 Iron–sulfur cluster biosynthesis

Conversion of L-cysteine into L-alanine by Csd releases sulfur that is incorporated into iron-sulfur clusters. Two additional cysteine desulfarases have been identified in *Synechocystis* but unlike Csd, neither are essential [90–92]. Iron–sulfur clusters are incorporated into many proteins involved in photosynthesis, respiration and nitrogen fixation [93]. Figure 4 outlines iron–sulfur biosynthesis (highlighted in green) and subsequent transfer to proteins, based on characterization of proteins in other bacterial species [94]. SufE acts as a sulfur donor, and IscA as a Fe^2+^ donor to the scaffold proteins required for cluster formation (SufA/NifU) [95]. Additional subunits (SufB/SufC/SufD) aid in transfer of the iron–sulfur cluster to proteins. NifU is possibly involved in repairing iron–sulfur clusters in proteins but has not been characterized in cyanobacteria.

## 6. Nucleotide biosynthesis

**Figure 5:**
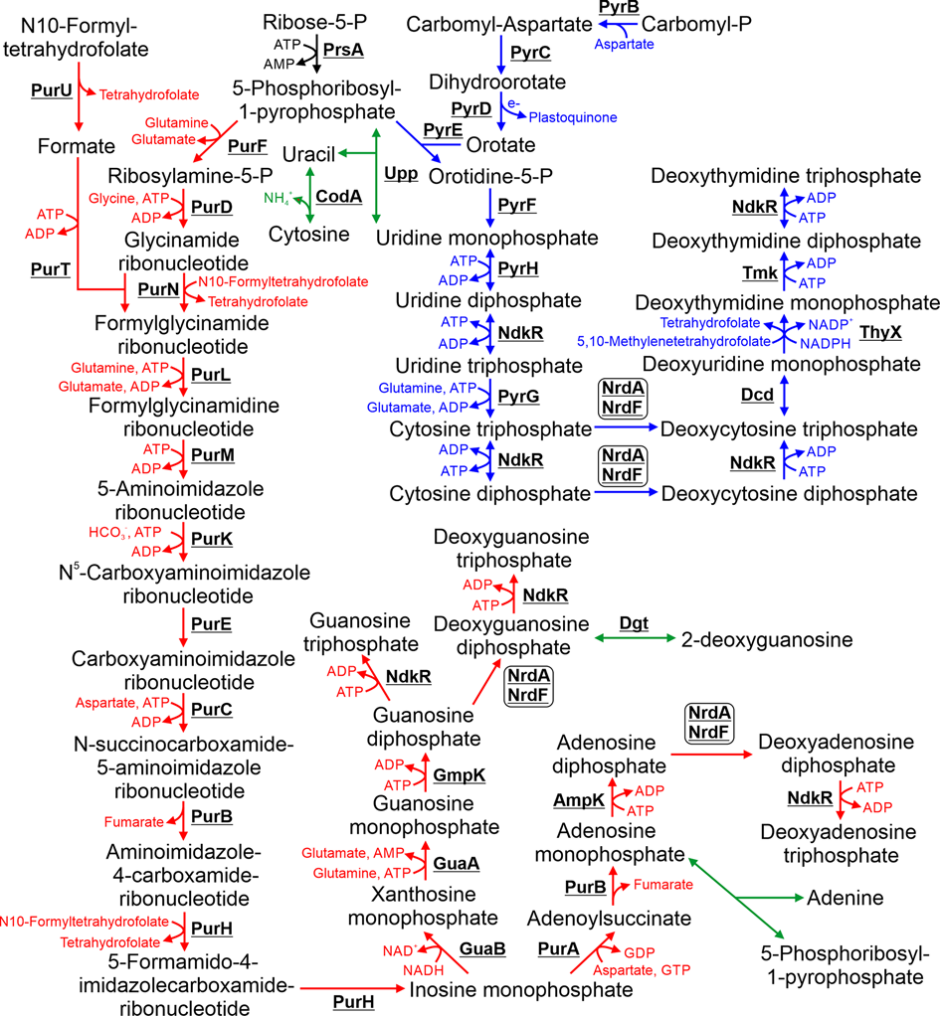
Metabolism of nucleotides The purine and pyrimidine biosynthesis pathways are highlighted in red and blue respectively. Possible nucleotide salvage pathways are highlighted in green. Cofactors in each reaction are shown with the exception of protons, water, oxygen and inorganic phosphate.

Enzymes involved in nucleotide biosynthesis (Figure 5) are highly conserved between *E. coli* and *Synechocystis* (Supplementary Table S1), and therefore this pathway has not been investigated in great detail in cyanobacteria. Pyrimidines and purines require the same precursor, 5-phosophoribosyl-1-pyrophosphate, which is synthesized from the PP pathway intermediate, ribose-5P, after which the pathways diverge.

### 6.1 Purine biosynthesis

In *E. coli*, purine biosynthesis requires eleven enzymatic steps for production of inosine monophosphate, the precursor of guanosine and adenosine based nucleotides (reviewed in [96]). *Synechocystis* encodes genes with high homology to all the purine biosynthetic enzymes required for inosine monophosphate in *E. coli*, including PurN and PurT, which are both capable of catalysing the third step (Supplementary Table S1). Both PurB and PurH catalyse two different steps in the pathway. In *E. coli*, inosine monophosphate is converted into guanosine diphosphate by GuaB, GuaA and GmpK, and adenosine diphosphate by PurA, PurB and AmpK [97]. All nucleoside-diphosphates are converted into nucleoside-triphosphates via NdkR [98] and to deoxyribonucleotides via the NrdA/NrdF complex [99]. All these enzymes are highly conserved between *E. coli* and *Synechocystis* (Supplementary Table S1).

### 6.2 Pyrimidine biosynthesis

In *E. coli*, pyrimidine biosynthesis requires six enzymatic steps for production of uridine diphosphate, the precursor of cytosine-, uridine- and thymidine-based nucleotides. Carbomyl-P, synthesized from glutamine and bicarbonate by CarA/CarB, is the initial substrate. Carbomyl-P is converted into orotate via a three-step pathway. Orotate phosophoribosyltransferase (PyrE) transfers a ribosyl group from 5-phosophoribosyl-1-pyrophosphate to orotate, forming oritidine-5-P, which is subsequently converted into uridine diphosphate via PyrF and PyrH. In *E. coli*, uridine diphosphate is converted into uridine triphosphate via NdkR, then cytosine triphosphate via PyrG [100]. The NrdA/NrdF complex then converts cytosine triphosphate into deoxycytosine triphosphate. The pathway for biosynthesis of deoxythymidine nucleotides has not been determined. However, enzymes homologous to those identified in the *Lactococcus lactis* pathway are conserved in *Synechocystis* [101]. Via this pathway, deoxycytosine triphosphate is converted into deoxyuridine monophosphate via Dcd, which is subsequently converted into deoxythymidine monophosphate via ThyX, which in turn is converted into deoxythymidine diphosphate via Tmk. However, experimental evidence is required to confirm whether this pathway is utilized by *Synechocystis*.

### 6.3 Nucleotide salvage pathways

*Synechocystis* also encodes a number of enzymes that display high sequence similarity to *E. coli* proteins involved in the nucleotide salvage pathway [100]. However, the role of the salvage pathway in cyanobacteria and how nucleotides are catabolized has not been investigated.

## 7. Cofactor biosynthesis

**Figure 6:**
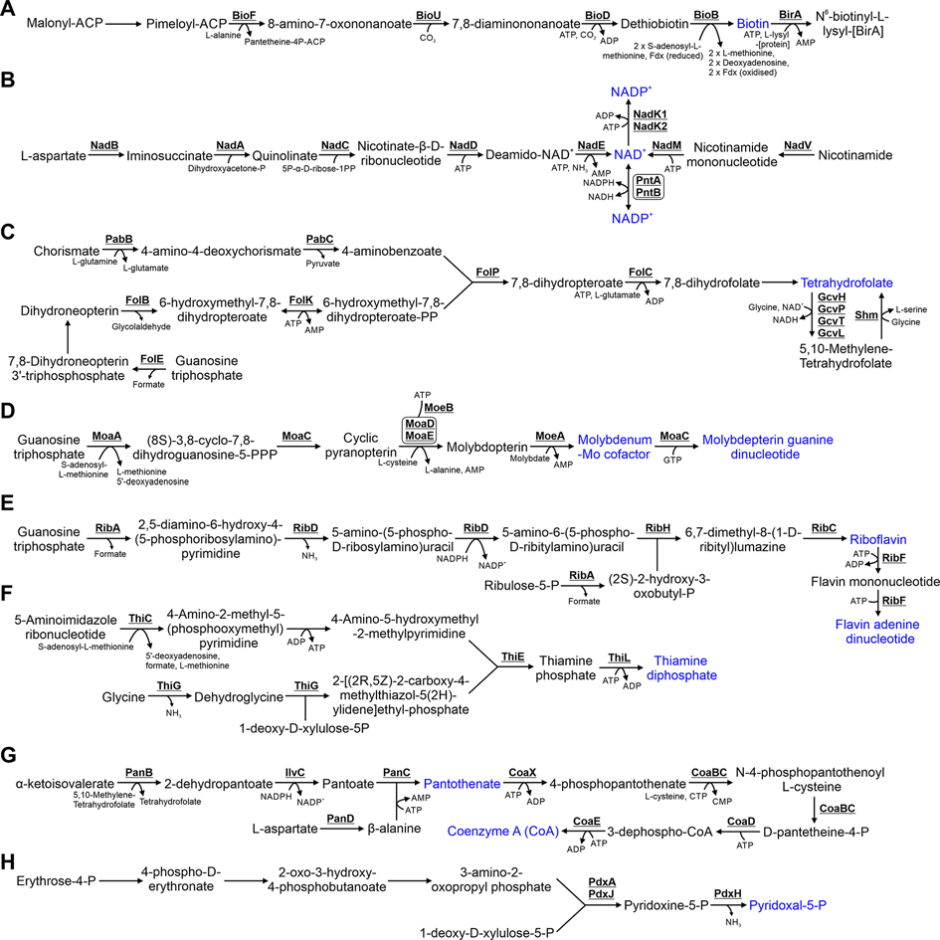
Metabolism of vitamins and cofactors Detailed are the pathways for biosynthesis of (**A**) Biotin, (**B**) NAD^+^ and NADP^+^, (**C**) folate, (**D**) molybdenum cofactors, (**E**) riboflavin and FAD, (**F**) thiamine, (**G**) pantothenate and coenzyme (**A** and** H**) pyridoxal-5P. Vitamins and cofactors are highlighted in blue. Cofactors in each reaction are shown with the exception of protons, water, oxygen and inorganic phosphate.

Unlike many cyanobacterial species, *Synechocystis* does not require the addition of any vitamins or cofactors for growth, suggesting that it encodes complete biosynthetic pathways for each essential compound. However, these pathways have not been extensively investigated. The majority of proteins in these pathways (Figure 6) have been assigned a function in cyanobacteria based on their homology to characterized enzymes from *E. coli*, with only a few enzymes characterized in *Synechocystis* or other model cyanobacterial species. Tocopherol biosynthesis is discussed in Section 9.4, since this cofactor is synthesized from the same initial substrates as other isoprenoids. Pseudocobalamin (Vitamin B_12_) biosynthesis is discussed in Section 10.4, since this cofactor is synthesized from the same initial substrates as bilins and chlorophyll.

### 7.1 Biotin biosynthesis

In *Synechocystis*, biotin (vitamin B_7_) is an essential cofactor required by acetyl-CoA carboxylase (AccA/AccB/AccC/AccD; Section 8.1), which is involved in fatty acid biosynthesis [102]. The biotin biosynthetic pathway has been determined in *E. coli* [103]. In *E. coli*, biotin is synthesized from malonyl-ACP-methyl ester, which undergoes two cycles of fatty elongation to form pimeloyl-ACP-methyl ester. This is subsequently converted to biotin via five enzymatic steps. Synthesis of the pimeloyl-ACP precursor has not been determined in *Synechocystis* [104]. Putative homologues of only three enzymes in the biotin biosynthetic pathway, BioF, BioD and BioB (and not BioH, BioC and BioA) are encoded in the *Synechocystis* genome (Figure 6A) [103]. Recently, a novel enzyme, BioU, was demonstrated to catalyse the same reaction as BioA, conversion of 8-amino-7-oxononoate to 7,8-diaminononanoate [105]. The enzymatic activity of BioU is different from BioA. BioU utilizes then reforms NADPH, consumes CO_2_, and acts as a suicide enzyme, meaning it catalyses only a single reaction due to loss of a lysine group. *Synechocystis* also encodes a putative BirA protein, which reacts with biotin to form a biotin–BirA complex that represses biotin biosynthesis [104].

### 7.2 NAD^+^ and NADP^+^ biosynthesis

Nicotinamide adenine dinucleotide (NAD^+^) is synthesized in cyanobacteria from L-aspartate by a five-step pathway encoded by most bacterial species (Figure 6B) [106]. The last two enzymes in the pathway, NadD and NadE, have low sequence similarity to the equivalent *E. coli* proteins but the activity of the enzymes has been confirmed in *Synechocystis* [107]. A second two-step pathway for NAD^+^ biosynthesis from nicotinamide has also been proposed [107,108], although how nicotinamide is produced has not been determined. NAD^+^ is converted into NADP^+^, required as an electron acceptor in linear photosynthetic electron transport, by NAD kinases, of which two are present in *Synechocystis* (NadK1, NadK2) [109]. The NAD^+^/NADP^+^ ratio is regulated by pyridine nucleotide transhydrogenase (PntA/PntB), which catalyses electron transfer between the two compounds [110].

### 7.3 Folate biosynthesis

Folate (vitamin B_9_) based cofactors (e.g. tetrahydrofolate, 5-methyl tetrahydrofolate, 5,10-methylene tetrahydrofolate) are required in certain enzymatic reactions for biosynthesis of the amino acids L-methionine, L-serine and glycine (Figure 4), the cofactors pantothenate and coenzyme A (Figure 6G), purine nucleotides and thymidylate pyrimidines (Figure 5) and certain tRNAs [111]. Folate is synthesized from the precursors, chorismate and guanosine triphosphate (Figure 6C). A two-step pathway (PabB/PabC) results in conversion of chorismate to 4-aminobenzoate. A four-step pathway (FolE/FolB/FolK and possible FolQ) catalyses the conversion of guanosine triphosphate to 6-hydroxymethyl-7,8-dihydropteroate-PP, which together with 4-aminobenzoate, catalyses the formation of 7,8-dihydropteroate. FolQ (Designated as NudB in *E. coli*) [112] has not been characterized in *Synechocystis* but slr0920 shows low sequence similarity to NudB (*e* value = 4.56E-06) and may perform FolQ enzymatic activity (Supplementary Table S2). 7,8-Dihydropteroate is subsequently converted into the different folate variants, although only one enzyme catalysing these steps, FolC, has been identified. Whether 5-methyl tetrahydrofolate is synthesized by *Synechocystis* is unknown, since the genome does not encode MetF, which synthesizes this compound from 5,10-methylene tetrahydrofolate in *E. coli* [111].

### 7.4 Molybdenum cofactor biosynthesis

Molybdenum cofactors (molybdopterin guanine dinucleotide or molybdopterin-Mo) act as catalytic centres in a range of enzymes. In *Synechococcus*, a molybdenum cofactor is required for nitrate reductase (NarB; Section 11.1) activity [113]. If any other enzymes in cyanobacteria also require molybdenum cofactors has not been determined. Molybdenum cofactors are synthesized from guanosine triphosphate (Figure 6D). This pathway has been characterized in *E. coli* and proteins demonstrating high sequence similarity to each enzyme have been identified in *Synechocystis* [113]. Moreover, several enzymes in the pathway have been characterized in *Synechococcus* [113,114]. MoaC is likely a bifunctional enzyme catalysing the second step, formation of pyranopterin, and the fifth step, synthesis of the cofactor molybdopterin guanine dinucleotide. The third step, conversion of cyclic pyranopterin to molybdopterin is catalysed by MPT synthase (MoaD/MoaE), which is regenerated by MoeB [115].

### 7.5 Riboflavin and flavin adenine dinucleotide biosynthesis

Riboflavin (vitamin B_2_) and flavin adenine dinucleotide (FAD) are also synthesized from guanosine triphosphate (Figure 6E). In cyanobacteria, FAD is a cofactor involved in flavoprotein-mediated redox reactions. The pathway is similar between *E. coli* and *Synechocystis* and enzymes are highly conserved between the species (Supplementary Table S1). Three enzymes, RibA, RibD and RibF, catalyse two separate reactions in the pathway.

### 7.6 Thiamine biosynthesis

Thiamine diphosphate (vitamin B_1_) is a cofactor for several enzymes, including pyruvate dehydrogenase (Section 3.1), transketolase in the OPP/CBB pathways (TktA, Section 3.2), and acetolactate synthase, catalysing the first step of L-valine, L-leucine and L-isoleucine biosynthesis (IlvG/IlvN; Section 5.1) [116]. It is synthesized from the purine biosynthetic intermediate, 5-aminoimidazole ribonucleotide (Section 6.1; Figure 5), glycine and 1-deoxy-D-xylulose-5-P (Figure 6F). The pathway has been largely characterized in *E. coli* [117], but in *Synechocystis*, homologues have not been identified for every protein in the pathway (Supplementary Table S1). Notably, there is no protein in *Synechocystis* with high sequence similarity to ThiD (Supplementary Table S2), which catalyses the second biosynthetic step starting at 5-aminoimidazole ribonucleotide.

### 7.7 Pantothenate and coenzyme A biosynthesis

The majority of enzymes involved in biosynthesis of pantothenate (vitamin B_5_; Figure 6G) and coenzyme A are highly conserved between *E. coli* and *Synechocystis* (Supplementary Table S1). Coenzyme A is required for formation of acetyl-CoA and in fatty acid biosynthesis. Three enzymes convert α-ketoisovalerate, an intermediate required for L-valine and L-leucine biosynthesis (Section 5.1; Figure 4), to pantothenate. An additional enzyme, PanD, catalyses the third step, conversion of L-aspartate to β-alanine [118]. The second reaction can be catalysed by PanE, not encoded in the *Synechocystis* genome (Supplementary Table S2) or IlvC, which is also involved in L-isoleucine, L-valine and L-leucine biosynthesis (Figure 4). Coenzyme A is synthesized from pantothenate via five enzymatic steps [118]. Only the first step (conversion of pantophenate to 4-phosphopantophenate) is catalysed by a different enzyme from that in the *E. coli* pathway, specifically a type III pantophenate kinase (CoaX) [119].

### 7.8 Pyridoxal-5P biosyntheis

Pyridoxal-5-P (vitamin B_6_) is a cofactor required by a range of enzymes involved in amino acid biosynthesis and catabolism, iron, cell wall component and carbon metabolism, and biosynthesis of other cofactors (For a full list refer to [120]). Biosynthesis of pyridoxal-5-P in *E. coli* utilizes 1-deoxy-D-xylulose-5-P and 3-amino-2-oxopropyl phosphate as substrates, and is catalysed via PdxA/PdxJ, then PdxH (Figure 6H) [121]. PdxA, PdxJ and PdxH are conserved in *Synechocystis* but the three enzyme pathway for 3-amino-2-oxopropyl phosphate biosynthesis has not been determined.

## 8. Membrane and cell wall biosynthesis

Cyanobacterial membrane composition differs from that of heterotrophic bacteria. Five classes of lipids accumulate in *Synechocystis* plasma and thylakoid membranes: phosphatidylglycerol, monogalactosyl-diacylglycerol, digalactosyl-diacylglycerol, sulfoquinovosyl-diacylglycerol and hydrocarbons [122,123]. Like other Gram-negative prokaryotes, cyanobacteria are encompassed by a peptidoglycan layer and an OM containing lipopolysaccharides (LPSs).

### 8.1 Lipid biosynthesis

**Figure 7:**
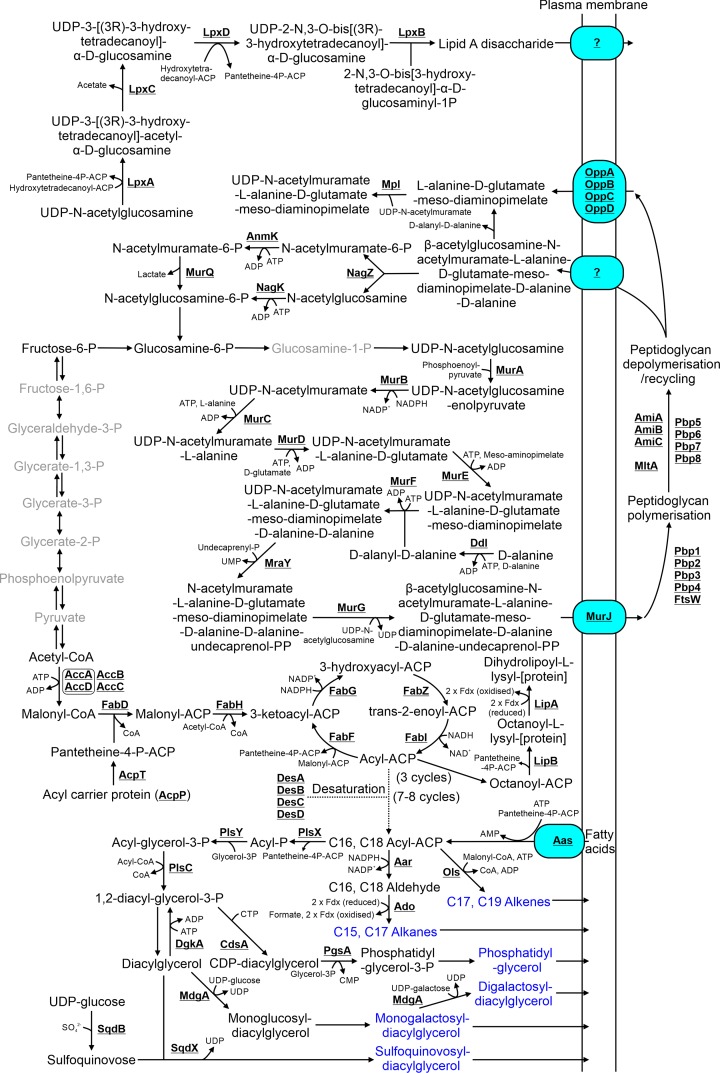
Metabolism of membrane lipids, peptidoglycan and lipopolysaccharides Membrane lipids are highlighted in blue. Steps highlighted in grey are compounds and reactions not involved in these pathways but detailed in Figure 2 and Figure 3. Cofactors in each reaction are shown with the exception of protons, water, oxygen and inorganic phosphate.

Cyanobacterial lipids are synthesized from acyl-ACPs (acyl carrier proteins), which in turn are synthesized from acetyl-CoA by a pathway similar to that in *E. coli* (Supplementary Table S1; Figure 7). Predominantly C16 and C18 acyl-ACPs are synthesized with various degrees of saturation catalysed by four possible desaturases (DesA, DesB, DesC, DesD) [124]. A PM associated protein, Aas [32], mediates import of acyl-ACPs and fatty acids from the PM and periplasm [125,126].

Hydrocarbons are synthesized directly from acyl-ACPs [127,128], with the majority of cyanobacteria (including *Synechocystis*) producing C15 or C17 alkanes via a two-step pathway (Aar/Ado) [129], while the remainder produce C17 or C19 alkenes via a polyketide synthase (Ols) [130]. The other lipids are synthesized from 1,2-diacyl-glycerol-3-P, which is produced from acyl-ACPs via three enzymes (PlsX, PlsY, PlsC) [131]. A further three enzymatic steps are required for phosphatidylglycerol biosynthesis. The enzyme catalysing the second step, PgsA, is non-essential in *Synechocystis*, when the mutant is supplemented with phosphatidylglycerol [132]. There is no *Synechocystis* protein with any sequence similarity to PgpB, the enzyme in *E. coli* that catalyses the third step (Supplementary Table S2).

1,2-Diacyl-glycerol-3-P is likely converted into diacylglycerol, the common substrate for synthesis of the other membrane lipids. The enzyme catalysing this step has not been identified. The reverse reaction is likely catalysed by DgkA. MgdA catalyses conversion of diacylglycerol to monoglucosyl-diacylglycerol, which is likely converted into monogalactosyl-diacylglycerol by an unidentified epimerase [133]. Monogalactosyl-diacylglycerol is then converted into digalactosyl-diacylglycerol by DgdA [134]. Sulfoquinovose, synthesized from UDP-glucose and sulfate by SqdB [135,136], is reacted with diacylgycerol by SqdX to form sulfoquinovosyl-diacylglycerol [137].

The *Synechocystis* genome encodes no proteins with homology to enzymes involved in β-oxidation (Supplementary Table S2), although one report has suggested that the capacity for fatty acid catabolism is retained [138]. If so, there must be an alternate, uncharacterized pathway responsible for lipid degradation.

### 8.2 Lipoic acid biosynthesis

Lipoic acids are cofactors required for a range of enzymes, including pyruvate dehydrogenase and the glycine cleavage system (GcvH/GcvP/GcvT/GcvL; Figure 2) [139]. The biosynthestic pathway has been elucidated in *E. coli* [140]. Lipoic acids are covalently attached to enzymes via LipB and then sulfonated via LipA. In contrast to *E. coli*, there are two putative LipA proteins in *Synechocystis* (Supplementary Table S1).

### 8.3 Peptidoglycan biosynthesis and depolymerization

The structure of *Synechocystis* peptidoglycan has not been determined. However, peptidoglycan in the closely related species, *Synechocystis* sp. PCC 6714, incorporates L-alanine, D-alanine, D-glutamate and meso-diaminopimelate into peptide bridges, which are linked to polymers consisting of alternating acetylglucosamine and acetylmuramate monomers. The enzymes synthesizing peptidoglycan monomers (acetylglucosamine-N-acetylmuramate-pentapeptides) from UDP-N-acetylglucosamine are highly conserved between *E. coli* and *Synechocystis* (Supplementary Table S1). Surprisingly, the last two enzymes in the pathway, MraY and MurG have been localized to the TM in *Synechocystis* [31,32], suggesting that an additional protein or process must transport these monomers to the PM. The flippase involved in translocating the acetylglucosamine-N-acetylmuramate-pentapeptide monomers to the periplasmic side of the PM in *E. coli* (MurJ) has not been identified in cyanobacteria [141]. However, the protein encoded by slr0488 in *Synechocystis* demonstrates some sequence similarity to MurJ (*E* value = 1.06E-28; Supplementary Table S1) but its function needs to be confirmed experimentally.

Polymerization of peptidoglycan is catalysed by the penicillin-binding proteins (PBPs) 1–4 and FtsW [142], while depolymerization and recycling of peptidoglycan monomers is catalysed by PBPs 5-8 and AmiA-C [143]. Four proteins in *E. coli* have been implicated in importing depolymerized peptidoglycan components (NagE, MurP, AmpG, Opp) [144], but only Opp, an oligopeptide transporter consisting of four subunits, is encoded in the *Synechocystis* genome (Supplementary Table S2). A series of cytosolic enzymes conserved in *Synechocystis* (Mpl, NagZ, AnmK, NagK, MurQ) likely recycle depolymerized peptidoglycan components back into peptidoglycan biosynthesis [144]. Other *E. coli* enzymes involved in recycling (NagA, NagB, AmiD, AmpB) have no homologues in *Synechocystis* (Supplementary Table S2).

### 8.4 Lipopolysaccharide biosynthesis

LPSs are incorporated into the OM of cyanobacteria, including *Synechocystis* [66]. Four enzyme synthesize the Lipid A disaccharide core of the LPS and are highly conserved between *E. coli* and *Synechocystis* (Supplementary Table S1). The protein involved in translocating Lipid A disaccharide to the periplasmic side of the PM has not been identified, although four PM localized proteins with high sequence similarity to MsbA (slr2019: *E* value = 8.64E-91; sll1276: *E* value = 2.28E-84; sll1725: *E* value = 7.22E-83; slr1149: *E* value = 1.82E-73; Supplementary Table S2), the characterized Lipid A disaccharide flippase from *E. coli* [145], are encoded in the *Synechocystis* genome [32]. Biosynthesis of the polysaccharide portion of the LPS has not been determined in cyanobacteria [146]. Five PM-localized glycosyltransferases are encoded by the *Synechocystis* genome which may play a role in saccharide polymerization (Supplementary Table S1). However, the *Synechocystis* genome encodes no proteins with homology to those in *E. coli* involved in transporting polysaccharides across the PM (i.e. Wzm/Wzt or Wzx), ligation of the polysaccharide to the Lipid A disaccharide core (WaaL) or transport of the fully synthesized LPS to the OM (LptA, LptC, LptD, LptE), with the possible exception of LptB (Supplementary Table S2).

## 9. Isoprenoid, quinol and carotenoid biosynthesis

Isoprenoids play a key role in electron transport, photoprotection, light harvesting, membrane integrity and organization, and are incorporated into a range of compounds including LPSs, peptidoglycan and chlorophyll.

### 9.1 Isoprenoid biosynthesis

**Figure 8:**
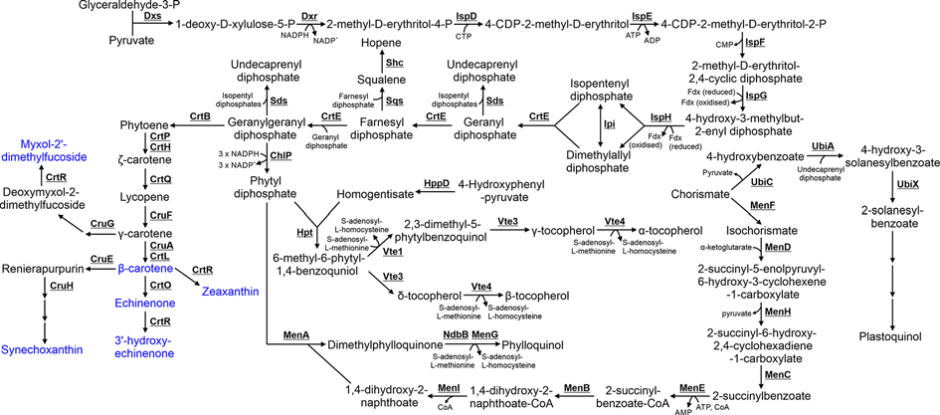
Metabolism of isoprenoids, quinols and carotenoids Carotenoids are highlighted in blue. Cofactors in each reaction are shown with the exception of protons, water, oxygen and inorganic phosphate.

Isoprenoids, specifically undecaprenyl diphosphate, farnesyl diphosphate and geranylgeranyl diphosphate, are substrates required for biosynthesis of a wide range of compounds including hopenes, LPSs, peptidoglycan, carotenoids, phylloquinone, plastoquinone, chlorophyll and tocopherols. Geranylgeranyl diphosphate is synthesized from pyruvate and glyceraldehyde-3-P via eight enzymes, all of which are highly conserved between *E. coli* and *Synechocystis* (Supplementary Table S1; Figure 8) [147]. An additional enzyme, Ipi, is involved in isomerization of isopentenyl diphosphate and dimethylallyl diphosphate [148]. *Synechocystis* mutants lacking Ipi demonstrate deficient isoprenoid biosynthesis, smaller cell size and reduced TMs, and an altered cell wall [149].

### 9.2 Hopene biosynthesis

Hopenes are synthesized from farnesyl diphosphate in *Synechocystis* via two enzymes: Sqs and Shc [150]. While the exact role of hopenes has not been determined in cyanobacteria, they have been suggested to play a role in membrane integrity in non-sulfur purple photosynthetic bacteria [151]. Hopenes have been detected in the TM, PM and OM of *Synechocystis* sp. PCC 6714 [152]. Sqs and Shc are expressed under photoautotrophic conditions in *Synechocystis* [32].

### 9.3 Carotenoid biosynthesis

Geranylgeranyl diphosphate is the substrate for carotenoid biosynthesis. Carotenoids play a key role in assembly of photosynthetic complexes [153], membrane integrity and thylakoid organization [154], and as light harvesting and photoprotective pigments. Seven carotenoids have been detected in *Synechocystis*: synechoxanthin, myxol-2′-dimethylfucoside (myxoxanthophyll), zeaxanthin, 3′-hydroxy-echinenone, *cis-*zeaxanthin, echinenone and β-carotene [155]. The pathway has not been completely elucidated [156–158], but 12 enzymes have been demonstrated to play a role in carotenoid biosynthesis.

### 9.4 Tocopherol biosynthesis

Tocopherols (Vitamin E) play a role in protecting cyanobacteria from lipid peroxidation [159], cold tolerance [160] and potentially optimizing photosynthetic activity [161]. All tocopherols are synthesized from the precursor 6-methyl-6-phytyl-1,4-benzoquinol, which is synthesized by Hpt utilizing the substrates phytyl diphosphate and homogentisate [162–164]. Phytyl diphosphate is synthesized from geranylgeranyl diphosphate by ChlP [165]. Homogentisate is synthesized from 4-hydroxyphenyl-pyruvate [166], which is typically synthesized from prephenate by TyrA. However, *Synechocystis* TyrA demonstrates specificity only to arogenate [167], suggesting that 4-hydroxyphenyl-pyruvate may be synthesized by an alternate route. Four tocopherols (α, β, δ, γ) are produced by *Synechocystis* [168], although it has not been determined if each has separate roles in the cell. α- and γ-Tocopherols are synthesized from 6-methyl-6-phytyl-1,4-benzoquinol via VTE1, VTE3 and VTE4, while β and δ tocopherols are synthesized via VTE3 and VTE4 [169].

### 9.5 Phylloquinone and plastoquinone biosynthesis

Phylloquinone (Vitamin K_1_) and plastoquinone are synthesized from chorismate. Phylloquinone acts as an electron acceptor in photosystem I [170], and while not essential under photoautotrophic conditions, loss of this compound results in a severe growth defect when cells are exposed to high light conditions [171]. Phylloquinone is synthesized by ten enzymes of which several have been characterized in *Synechocystis* [171,172]. The majority have been identified based on homology with proteins synthesizing menaquinone (Vitamin K_2_) and characterized in other bacteria [173]. The second last enzyme in the pathway, MenA, utilizes phytyl diphosphate, while the last enzyme requires that dimethylphylloquinone be reduced via NAD(P)H dehydrogenase NdbB to dimethylphylloquinol, prior to synthesis of phylloquinone by MenG [174].

Plastoquinone is an essential electron carrier required for photosynthesis and respiration [23]. Despite the importance of plastoquinone, the entire biosynthetic pathway has not been determined [175]. Catalytic activity of only the first three enzymes in the pathway, UbiC, UbiA and UbiX, has been determined by expression of the *Synechocystis* genes in *E. coli* [175,176]. Deletion of a putative 4-hydroxy-3-solanesylbenzoate decarboxylases, encoded by *sll0936*, results in reduced plastoquinone levels [175], suggesting an uncharacterized role for this protein in its biosynthesis.

## 10. Chlorophyll, phycobilin and pseudocobalamin biosynthesis

Chlorophyll and phycobilins are the light harvesting pigments incorporated into photosystems and phycobilisomes, respectively. Pseudocobalamin (vitamin B_12_) is synthesized from the same precursor substrate, uroporphyrinogen III, and is therefore included in this section.

### 10.1 Haem biosynthesis

**Figure 9:**
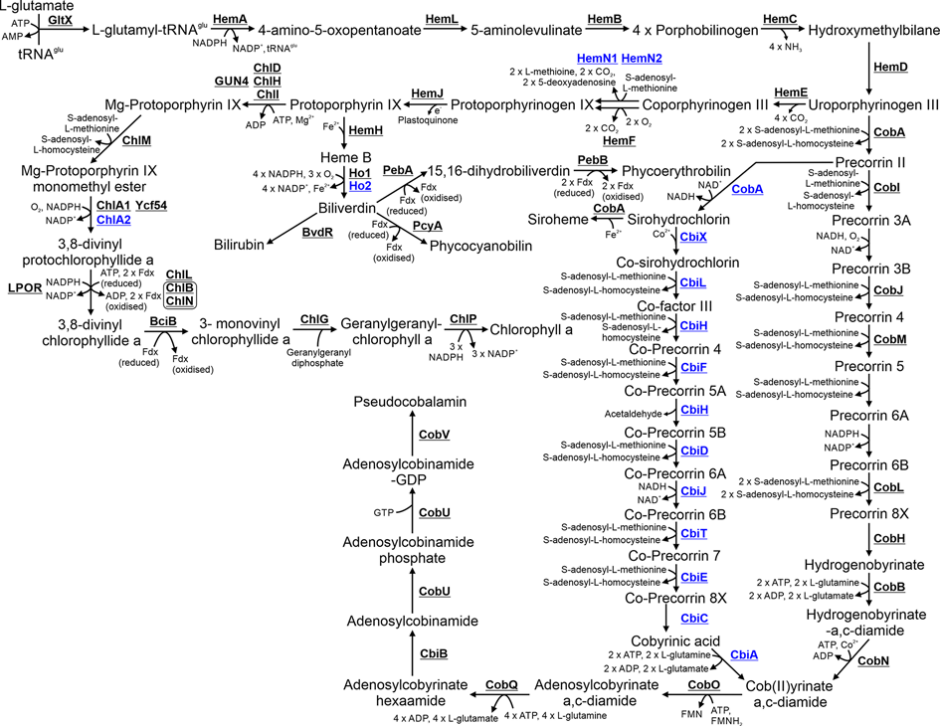
Metabolism of chlorophyll, phycobilin and pseudocobalamin Proteins involved in anaerobic or low oxygen environment enzymatic steps are highlighted in blue. Cofactors in each reaction are shown with the exception of protons, water and inorganic phosphate.

Haem, the precursor of phycobilins, is synthesized from L-glutamate and tRNA^Glu^ via ten enzymatic steps (Figure 9). All enzymes, apart from HemJ, are highly conserved between *E. coli* and *Synechocystis* (Supplementary table 1) [177]. In contrast with *E. coli*, HemJ, not HemG or HemY, is the protophyrinogen IX oxidase most commonly found in cyanobacteria [178]. HemJ likely requires plastoquinone as an electron acceptor in *Chlamydomonas reinhardtii* [179] and localization of *Synechocystis* HemJ to the TM [32] suggests a similar enzymatic reaction. The *Synechocystis* genome also encodes additional enzymes expressed under micro-oxic conditions, including HemN1 (and possibly HemN2) [180], which can catalyse the eighth enzymatic step of haem biosynthesis, in addition to Ho2 [181,182] and ChlA2 [183], which are involved in bilin and chlorophyll biosynthesis, respectively. It should be noted that these enzymes still require oxygen for catalytic activity. However, they may bind oxygen with greater affinity than the enzymes catalysing the same step that are expressed under non-microoxic conditions. Haem does not accumulate in mutants deficient in Ho1 and Ho2, which catalyse the first steps in bilin biosynthesis, suggesting that haem is rapidly degraded by an uncharacterized pathway [182].

### 10.2 Bilin biosynthesis

Haem is the substrate for biosynthesis of biliverdin, which in turn is the substrate for production of the pigments phycocyanobilin and phycoerythrobilin. These pigments are subsequently incorporated into the light harvesting phycobilisome complex [184]. *Synechocystis* only produces phycocyanobilin via the enzyme PcyA [184]. *Synechocystis* also encodes a biliverdin reductase, BvdR, resulting in production of bilirubin [185]. While the exact role of bilirubin has not been determined, deletion of BvdR results in a mutant with severely attenuated phycobilisomes.

### 10.3 Chlorophyll biosynthesis

Chlorophyll, the main pigment in photosystems, is synthesized from protoporphyrin IX, the immediate precursor of haem, via seven enzymatic steps. The complete pathway has been characterized in *Synechocystis*. The first step of chlorophyll biosynthesis is catalysed by three magnesium chelatase enzymes, ChlD, ChlH and ChlI, resulting in production of Mg-protoporphyrin IX [186]. GUN4 is also essential for magnesium chelatase activity [187–189]. The second step is catalysed by ChlM [190], while the third is catalysed via ChlA1 (AcsF) or ChlA2 [191]. Ycf54 may also be required for ChlA1 activity [192]. Two independent enzymes, a light-dependent NADPH:protochlorophyllide reductase (LPOR) or a ferredoxin-dependent DPOR complex, can catalyse the following step [193], while BciB catalyses the step after this [194,195]. Geranylgeranyl is incorporated into chlorophyll by ChlG in the second last step. In a landmark paper, expression of ChlDHI and GUN4, ChlM, ChlA1, LPOR, BciB, ChlG and ChlP in *E. coli* was sufficient for chlorophyll biosynthesis [196], demonstrating that no other enzymes are required in this pathway.

### 10.4 Pseudocobalamin biosynthesis

Cobalamin (Vitamin B_12_) is required for activity of MetH, involved in methionine biosynthesis (Figure 4), and may be required by certain enzymes in the quinone and folate biosynthesis pathways [197]. Cyanobacteria produce an alternate form of vitamin B_12_ termed pseudocobalamin [198]. Vitamin B_12_ is synthesized from the haem biosynthetic intermediate, uroporphyrinogen III. The cob(II)yrinate a,c-diamide component of vitamin B_12_ can be synthesize by either an aerobic or anaerobic pathway, which share certain enzymes [199]. These pathways have been characterized in a range of heterotrophic bacteria [199,200] but relatively few cyanobacterial enzymes have been investigated. *Synechocystis* encodes all the enzymes in the anaerobic pathway but is missing five in the aerobic pathway (CobG, CobF, CobK, CobS, CobT), suggesting that this biosynthetic route is not utilized (Supplementary Table S1). Several enzymes involved in converting cob(II)yrinate a,c-diamide to pseudocobalamin (CobO, CobQ, CbiB, CobU, CobV) are potentially encoded in the *Synechocystis* genome. However, the exact biosynthetic steps have not been determined and the pathway in *Synechocystis* can only be speculated based on characterized pathways from species that synthesize cobalamin [199].

*Synechocystis* also has the genetic potential to produce siroheme from the pseudocobalamin biosynthetic intermediate, sirohydrochlorin. Siroheme is a cofactor required for nitrite reductase [201] and possibly for other enzymes.

## 11. Transport systems

**Figure 10:**
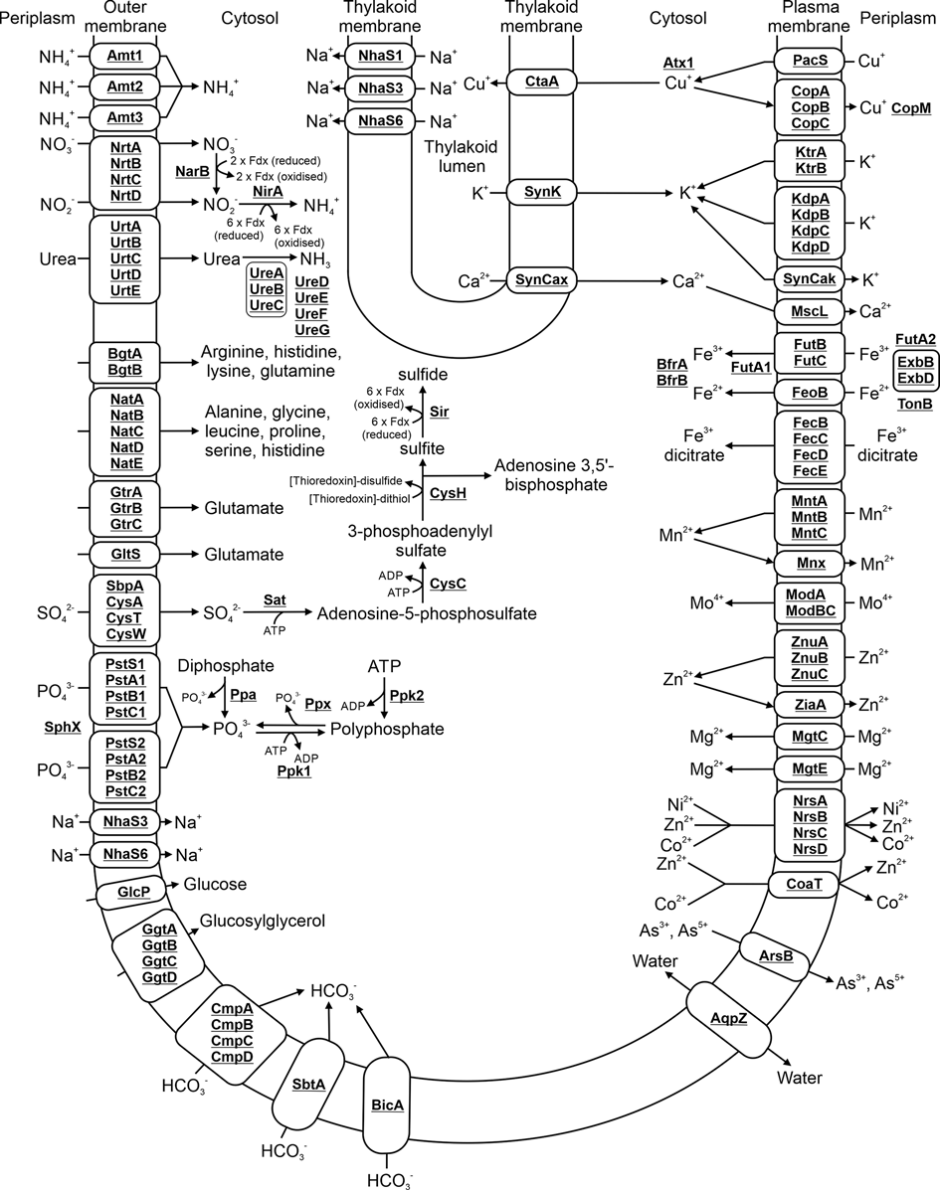
Proteins involved in metabolite transport and conversion of nitrogen, sulfur and phosphate based compounds Localization of transporters in either the PM or TM is detailed. Subunits in each complex may not all be membrane localized but soluble. Cofactors in each reaction are shown with the exception of protons, water, oxygen and inorganic phosphate.

The majority of proteins potentially involved in metabolite transport localize to the PM (Figure 10) [32]. However, there are many putative transporters in *Synechocystis* with no assigned function (Supplementary Table S4), suggesting that our knowledge of cyanobacterial metabolite transport is still incomplete.

### 11.1 Ammonia, nitrate, nitrite and urea transport

A range of transporters are responsible for import of nitrogen-based compounds. *Synechocystis* encodes three ammonium transporters (Amt1, Amt2, Amt3), with Amt1 being responsible for the majority of uptake [202]. Another transporter complex, comprising four subunits, NrtA-D, imports nitrate and nitrite [203–205]. Nitrate is reduced to nitrite by NarB [206], while NirA converts nitrite to ammonium [207]. Both enzymes require electrons supplied by ferredoxin [201]. *Synechocystis* can also utilize urea, which is imported into the cell via a transporter complex composed of five subunits, UrtA-E [208]. Urea is converted into two molecules of ammonia via the urease complex comprising three subunits, UreA-C, which is assembled by four accessory proteins, UreD-G [209].

### 11.2 Amino acid transport

A range of permeases with affinity for different amino acids have been characterized in *Synechocystis* in an extensive study conducted by Quintero et al [210]. The basic amino acid transporter encoded by BgtA and BgtB mediates transport of L-arginine, L-histidine, L-lysine and L-glutamine. Two transporters, the Gtr complex composed of GtrA-C, and the single protein GltS system, mediate L-glutamate transport. The neutral amino acid system encoded by NatA-E mediates transport of L-alanine, glycine, L-leucine, L-proline, L-serine and L-histidine. A separate study also implicated this transporter in import of L-cysteine [211]. Whether these transporters can export amino acids or transport any of the other ten amino acids is unknown. It is also possible that uncharacterized permeases may play a role in transport of other amino acids.

### 11.3 Metal ion transport

The *Synechocystis* genome encodes a range of transporters mediating import of metal ions into the cytosol, and in the case of Cu^+^, into the thylakoid lumen. Additional transporters are also required for metal homeostasis and efflux.

#### 11.3.1 Copper transport

Three copper (Cu^+^) transporters, CtaA, PacS and the Cop complex, have been characterized in *Synechocystis*. Cyanobacteria require Cu^+^ in the thylakoid lumen for the electron carrier plastocyanin. Proteome mapping of *Synechocystis* localized PacS to the PM and CtaA to the TM [32], suggesting these are the main Cu^+^ importers in each membrane [212]. A chaperone, Atx1, likely localizes to the cytosol but possibly also to the thylakoid lumen, binds Cu^+^ and delivers it to proteins requiring it for enzymatic activity [213,214]. The Cop complex, composed of CopA-C, is involved in Cu^+^ efflux [215]. An additional protein, CopM, binds Cu^+^ in the periplasm and mutants lacking this protein are highly sensitive to elevated levels of Cu^+^ [216].

#### 11.3.2 Potassium transport

*Synechocystis* encodes two PM localized potassium (K^+^) uptake systems, Ktr (KtrA/KtrB) and Kdp (KdpA, KdpB, KdpC, KdpD) [217]. The Ktr system mediates rapid K^+^ uptake while the Kdp system maintains K^+^ levels under limiting conditions in the environment [217,218]. KtrC was initially incorrectly assigned as a subunit of the Ktr complex [219], but was later assigned to monoglucosyldiacylglycerol synthesis, not K^+^ import [134]. A third TM localized transporter, SynK [220], is responsible for K^+^ efflux from the thylakoid lumen [221]. An additional calcium activated, PM localized transporter, SynCak, may also be involved in potassium transport [222]. Deletion of SynCak in *Synechocystis* results in a mutant with altered membrane potential and greater resistance to zinc.

#### 11.3.3 Calcium transport

Calcium (Ca^2+^) transport is not well understood in cyanobacteria. A putative Ca^2+^/H^+^ antiporter, SynCax, has been identified [223,224], and localizes to the TM [32]. A PM localized Ca^2+^ importer has not been identified. MscL has been proposed to be involved in Ca^2+^ export [225].

#### 11.3.4 Iron transport

Iron is potentially imported into *Synechocystis* via multiple transporters, although only the Fut system is essential [226,227]. FeoB, which imports Fe^2+^, is the main iron transporter in *Synechocystis* [228]. In the Fut system, a periplasmic protein, FutA2, bind Fe^3+^ [229,230] prior to uptake by the FutB/FutC membrane transporter [227]. A second futA protein, FutA1, has been postulated to bind Fe^3+^ in the cytosol [228], although proteome mapping localized it to the PM [32]. Three ExbB–ExbD complexes identified in *Synechocystis*, possibly in association with TonB and one to three putative FhuA OM transporters, are also required for iron uptake [226,231]. Once imported, iron is stored in ferritin complexes (BfrA, BfrB) in the cytosol [232]. *Synechocystis* also encodes subunits of a putative Fe^3+^ dicitrate transporter, although this system is reportedly less important for iron import [104].

#### 11.3.5 Manganese, molybdate, zinc and magnesium transport

Manganese (Mn^2+^) is imported into *Synechocystis* via the MntABC complex [233], although other low-affinity transport systems may be present. Mn^2+^ plays a key role in the oxygen evolving centre of photosystem II. Mnx is essential for tolerance of *Synechocystis* to high manganese levels and may play a role in exporting Mn^2+^ from the cytosol to the thylakoid lumen [234]. The *Synechocystis* genome encodes proteins (ModA and ModBC) with high homology to the characterized molybdate transporter of *E. coli* (E values = 6.32E-37 and 9.94E-51, respectively) [235], but this complex has not been characterized in a cyanobacterium. The zinc (Zn^2+^) transporter, composed of the ZnuA, ZnuB and ZnuC subunits, is highly conserved between *E. coli* and *Synechocystis* (Supplementary Table S1). Only the ZnuA protein has been characterized in *Synechocystis* [236]. A separate protein, ZiaA, is involved in Zn^2+^ export [237]. Atx1 may also act as a Zn^2+^ chaperone, in addition to its role as a Cu^2+^ chaperone [238]. The *Synechocystis* genome also encode two putative magnesium transport proteins, MgtC and MgtE [239], both of which localize to the PM [32].

#### 11.3.6 Cation efflux systems

A number of cation efflux systems are encoded by the *Synechocystis* genome. The Nrs complex (NrsA, NrsB, NrsC, NrsD) was induced when cells were exposed to excess Ni^2+^, Co^2+^ and Zn^2+^, the CoaA transporter when cells were exposed to Co^2+^ and Zn^2+^, and the ArsB transporter by exposure to arsenic [240].

#### 11.3.7 Sulfate transport

Sulfate is transported into cells by the SbpA/CysA/CysW/CysT system, which is highly conserved between *E. coli* and *Synechocystis* (Supplementary Table S1). Sulfate is converted into sulphide by the assimilatory pathway divided into four enzymatic steps. The enzymes catalysing the final three steps are conserved between *E. coli* and *Synechocystis*. The first enzyme in the pathway, Sat, is widely conserved in bacteria capable of sulfate reduction.

#### 11.3.8 Phosphate transport

*Synechocystis* contains two systems for phosphate uptake, Pst1 and Pst2, each composed of four subunits [241,242]. The PstS subunits of each system, in addition to SphX, bind phosphate in the periplasm, prior to uptake [242]. Following uptake, phosphate can be stored in polyphosphate, which consists of polymers containing tens to hundreds of phosphates. Phosphate is converted into polyphosphate by polyphosphate kinase (Ppk1), via sequential addition of single residues [243]. A second Ppk enzyme, Ppk2, homologous to an enzyme characterized in *Pseudomonas aeruginosa* [244], likely synthesizes polyphosphate from ATP. Ppx catalyses depolymerization of polyphosphate, releasing inorganic phosphate [243]. Another enzyme, Ppa, converts diphosphate to phosphate and is essential in *Synechocystis* [243].

### 11.4 Sodium antiporters

*Synechocystis* encodes six putative sodium (Na^+^) antiporters [245], three of which localize to the TM (NhaS1, NhaS3, NhaS6) and two to the PM (NhaS2, NhaS5) [32]. Only NhaS3 is essential in *Synechocystis* [246]. NhaS3 has been suggested to play a role in maintaining not just H^+^ and Na^+^, but also K^+^ homeostasis [247]. Deletion of the remaining Nha antiporters did not affect growth, even when cells were exposed to high salt concentrations, suggesting that these proteins can compensate for loss of each other [246].

### 11.5 Organic and inorganic carbon transport

*Synechocystis* encodes transporters that import a range of organic carbon compounds. These include GlcP that imports glucose [36] and the Ggt complex, which imports glucosylglycerol and possibly sucrose and trehalose [248,249]. A number of transporters for inorganic carbon have been characterized in *Synechocystis*. These play a key role in the CO_2_-concentrating mechanism during photosynthesis, and include the Cmp complex (BCT1 transporter) [250,251], the SbtA transporter [252,253] and the BicA transporter [254].

### 11.6 Water transport

*Synechocystis* encodes an aquaporin water channel, aqpZ, which is required for regulating osmotic stress [255], and is essential for mixotrophic growth [256].

## 12. Future directions in understanding cyanobacterial metabolism

Gaining a complete understanding of cyanobacterial metabolism is dependent on optimizing the slow process of mutant generation and characterization, and developing bioinformatics tools that provide better insight into protein function, in order to easily develop enzyme assays. To bypass the laborious step of mutant generation, we are developing CyanoSource, a mutant library targeting every gene in *Synechocystis*. Construction of the library is outlined in Gale et al [257]. Building on our transformation and Modular Cloning (MoClo) techniques [258,259], we will collaborate with United Kingdom DNA Foundries in Norwich and Edinburgh to automate the generation of a whole genome library of gene insertion plasmids (representing 3456 coding sequences (CDSs)), and will transform *Synechocystis* to generate the largest available collection of known and novel cyanobacterial mutant strains.

Each CyanoSource plasmid will consist of a pUC19 based backbone into which two regions flanking the gene of interest will be inserted. Between these regions a positive selectable marker, a cassette conferring resistance to kanamycin (KanR), and a counter-selection negative selectable marker based on the cytosine deaminase protein CodA [260], will be inserted. Marked mutants will be generated by transformation of the plasmid into *Synechocystis* and growth of the mutant on increasing concentrations of kanamycin. If segregated mutants are not obtained on agar plates containing kanamycin concentrations of 400 μg/ml, the gene will be deemed essential. In this case, other growth conditions may be trialled, in addition to growth on different types of metabolites to generate auxotrophic mutants. Conditional mutants (i.e. specialized mutants that require an external stimulus to repress a gene) will be constructed for essential genes that cannot be removed by any of these mechanisms. Only marked mutants will be generated for CyanoSource. For generation of unmarked mutants, users can easily excise the kanR/CodA cassette and the plasmid containing just the backbone and flanking regions can be introduced into the marked mutant. Unmarked mutants are selected by growth of transformants on agar plates containing 5-fluorocytosine. CodA converts this chemical to 5-fluorouracil, which is highly toxic to many bacteria. All strains, including knockouts, partially segregated, conditional and auxotrophic mutants, and plasmids containing the flanking regions interspersed with the positive and negative selectable markers, will be made available to the academic and biotechnology community as these are constructed throughout 2020/21.

This library will allow us to determine the essential *Synechocystis* gene set, which can be compared with the one generated in *Synechococcus* via transposon mutagenesis [261]. This will provide insight into the essential gene set of the phylum. CyanoSource may also provide insights into the function of many proteins involved in metabolism. Generation of auxotrophic mutants will provide strong evidence that the encoded protein is involved in the same pathway as putative characterized homologues from other species. However, deletion of these genes may only be possible if the metabolite the encoded protein plays a role in synthesizing can be imported into the cell. Research groups with expertise in enzyme and pathway characterization but lacking expertise in generation of cyanobacterial mutants may also be encouraged to investigate the function and enzymatic activity of cyanobacterial proteins, especially in light of recent high-impact publications on characterization of *Synechocystis* enzymes and pathways [79,105].

A better understanding of *Synechocystis* metabolism will help to expand on current gaps in the metabolic biochemistry, as outlined in this review. Since it is likely that a high proportion of these pathways are conserved throughout the phylum, understanding *Synechocystis* metabolism will aid our understanding of cyanobacterial species that play a key role in the environment (e.g. marine *Prochlorococcus* and *Synechococcus* species) or which have characteristics ideal for biotechnology (e.g. the fast growing cyanobacteria, *Synechococcus* sp. PCC 11901 [262]). This will be critical in optimization of biotechnologically relevant species as renewable platforms for production of chemicals currently derived from fossil fuels.

## Supplementary Material

Supplementary Refs. Material

Supplementary Table S1

Supplementary Table S2

Supplementary Table S3

Supplementary Table S4

## Abbreviations

2-OGDC: α-ketoglutarate decarboxylase
CBB: Calvin–Benson–Bassham
OM: outer membrane
PBP: penicillin-binding protein
PM: plasma membrane
PP: pentose phosphate
SSADH: succinic semialdehyde dehydrogenase
TCA: tricarboxylic acid
TM: thylakoid membranes
