# Supplementary material for: Current knowledge and recent advances in understanding metabolism of the model cyanobacterium Synechocystis sp. PCC 6803
Source: Biosci Rep. 2020 Apr 3;40(4):BSR20193325. doi: 10.1042/BSR20193325 (PMC7133116; doi:10.1042/BSR20193325)
Supplement: Supplementary Refs. Material [file BSR-2019-3325C_supp.pdf]

263. Kanehisa M (2019) Toward understanding the origin and evolution of cellular organisms. *Protein Sci* 28(11):1947-1951.
264. Kanehisa M & Goto S (2000) KEGG: kyoto encyclopedia of genes and genomes. *Nucleic Acids Res* 28(1):27-30.
265. Kanehisa M, Sato Y, Furumichi M, Morishima K, & Tanabe M (2019) New approach for understanding genome variations in KEGG. *Nucleic Acids Res* 47(D1):D590-D595.
266. Altschul SF, Gish W, Miller W, Myers EW, & Lipman DJ (1990) Basic local alignment search tool. *J Mol Biol* 215(3):403-410.
267. UniProt C (2010) The Universal Protein Resource (UniProt) in 2010. *Nucleic Acids Res* 38(Database issue):D142-148.
